# Impact of 6-Week Combined Gym and Climbing Training on Handgrip Strength and Arm Size—GRIP-6 Study

**DOI:** 10.3390/jfmk10040427

**Published:** 2025-11-03

**Authors:** Tomasz Chomiuk, Adam Męczyński, Przemysław Kasiak, Artur Mamcarz, Daniel Śliż

**Affiliations:** 1 3rd Department of Internal Medicine and Cardiology, Medical University of Warsaw, 02-091 Warsaw, Poland; 2 Faculty of Health Sciences, Medical University of Warsaw, 02-091 Warsaw, Poland

**Keywords:** climbing, strength training, handgrip, muscle size, gym

## Abstract

**Background**: Climbing and strength training are among the most popular types of sports among recreational athletes. However, many newcomers quickly lose motivation and abandon training due to a lack of visible and athletic progress. Hence, we assessed whether a 6-week combined structured gym and climbing training could improve arm muscle strength and size. **Methods**: We recruited 25 healthy recreational athletes (14 [56.0%] females, age = 20.4 ± 1.7 years, BMI = 21.8 ± 1.7 kg·m^−2^). Most participants trained several times per week (N = 12, 48.0%), and training sessions lasted between 1 and 2 h (N = 11, 44.0%). Most athletes rated their current fitness level as “very good” (N = 15, 60.0%). Subjects performed two gym sessions and two climbing training sessions per week and avoided other training for 6 weeks. We measured the time of bar hang, handgrip strength, and forearm circumference before and after 6 weeks. **Results**: Hanging time increased from 55.3 ± 3.2 s to 60.9 ± 31.3 s (t(24) = 6.68, *p* < 0.001). Right handgrip strength increased from 31.6 ± 8.4 N to 34.3 ± 7.6 N (t(24) = 5.58, *p* < 0.001). Left handgrip strength increased from 29.4 ± 9.9 N to 31.0 ± 9.8 N (t(24) = 4.62, *p* < 0.001). Right forearm circumference increased from 24.6 ± 1.5 cm to 25.4 ± 1.7 cm (t(24) = 9.04, *p* < 0.001). Left forearm circumference increased from 24.6 ± 2.0 cm to 25.1 ± 2.1 cm (t(24) = 5.94, *p* < 0.001). All the relationships remained significant when stratified between males (*p* < 0.001–0.003) and females (*p* < 0.001–0.008). **Conclusions**: A 6-week training intervention consisting of structured climbing and strength training induces significant improvements in grip strength and the appearance of arm muscles in recreational athletes. Amateurs could benefit even from shorter training mesocycles, as a 6-week window to see visible progress.

## 1. Introduction

Climbing is a sport that involves moving on ice, rock, or other artificial structures, requiring the use of at least one hand [1]. This distinguishes it from mountaineering or sloping terrain, where the body typically relies on the feet to maintain balance [2]. Climbing varies greatly in terms of the type of muscle contractions, the nature of the work performed, and the type of physical exertion [1]. A crucial element is body positioning and forearm coordination during climbing, which places the least possible strain on the muscular system and promotes using greater loads at maximum-intensity conditions [3]. This depends on several factors, such as the degree of overhang of the route, specific grip position, and the individual skills of the climber [3,4]. However, the size and shape of the holds and muscle strength remain crucial [5].

Muscle function depends on the size and position of holds and the athlete’s body position [6]. Upper limb muscles and fingers are engaged during climbing and play the greatest role in climbing [7]. They develop significant strength by supporting the entire body in the hold. They often determine the duration of physical exertion due to their rapid fatigability [8]. The low endurance of their muscle fibers is due to the specificity of the climbing, which is a continuous, static effort [8]. Despite the small cross-sectional area of muscles in fingers and arms, the fibers are forced into prolonged contraction. The lower body muscles, such as knee extensors and plantar flexors, are also involved, but to a lesser degree [1].

Strength training has several benefits for health and is increasingly popular among amateurs and those starting their adventure with sports [9]. Amateurs often practice several sports, mixing them at different times to avoid burnout [10,11]. A common combination is strength training and rock climbing [5]. Practicing each type of sport will bring mutual benefits and improve performance in the other [5]. To enhance performance in climbing, strength training is essential to improve muscle strength [5]. Both strength and climbing training improve grip strength and muscle endurance [8]. Climbers often used strength training as their accessory work [5]. It has a positive impact on isometric, concentric, and eccentric work, which occur in climbing [12].

Typically, at least 8 weeks and, preferably, 12–16 weeks of systematic training are recommended to notice visible results [13,14]. However, amateurs are easily discouraged from training, and there is a need to find the minimal effective mesocycle to keep their engagement [11]. Previous studies reported that shorter duration of training plans is used and promise visible improvements in athletic performance (e.g., 6 weeks) [15]. However, the exact degree of improvement in athletic performance has been ambiguous, and the effect of specific paired strength and climbing training is still controversial, especially among recreational athletes [16,17].

Therefore, in this study, we assessed whether a 6-week combined structured gym and climbing training could already improve athletic performance and muscle appearance in amateur athletes.

## 2. Materials and Methods

### 2.1. Study Design

This study was reviewed and met the requirements of the Bioethical Committee of the Medical University of Warsaw (code AM#077109 from 15 December 2021) and all procedures followed Declaration of Helsinki. All the participants provided their written informed consent. It was a longitudinal study, and we reported the protocol according to STROBE guidelines for observational studies [18]. The checklist is attached in Supplementary Material S1. Measurements at baseline and after the 6-week protocol were conducted at the Centre of Sport and Rehabilitation of the Medical University of Warsaw (Medical University of Warsaw, Warsaw, Poland) between January 2022 and February 2022.

We recruited the participants via in-person meetings and personal inquiries. Inclusion criteria were as follows: (1) age ≥18 years, (2) no structured strength training ever in life, (3) no history of acute or chronic injuries, (4) no chronic or acute medication use, and (5) basic climbing training experience. We defined structured training as following a training plan and attending the gym three or more times per week. If a participant followed the structured gym training, the climber was not included in the study. We defined basic climbing experience as at least a month of regular training, at least once per week. Our goal was to recruit individuals with minimal experience in climbing but familiar with the specificity of this type of sport to test whether structured accessory strength training could produce a visible enhancement in climbing beginners. Participants may have participated in other sports in the past, but it could not have been gym training or climbing. They were instructed to avoid other sports during the experimental protocol to not overlapping the progress from various types.

### 2.2. Study Outcomes

We assessed basic demographic parameters (age, height, body weight, training experience, training regimen, health status) in an author’s survey (doubts or missing data were verified by the supervising researcher during the measurements). We assessed the grip strength, hang time, and forearm circumference. Participants received visual and verbal instructions regarding the bar hang and flexor strength measurements. Participants were familiar with the protocol and were verbally encouraged to achieve the best possible result. Each measurement was performed three times with a 3 min break between trials and averaged.

The hanging time on the bar was performed as follows. The subject positioned themselves under the bar, facing the examiner, with arms placed at shoulder-width and chin above the bar. Then, the subject grasped the bar, lifted their legs off the ground, and hung passively at straight arms until they fell. Participants were instructed to keep a dorsal grip on the bar. The handgrip test was measured on a hand dynamometer (Takei Scientific Instruments Co., Tokyo, Japan). To eliminate external forces and generate maximum grip force, subjects performed the tests in a sitting position and in the sagittal plane with wrists in a neutral position. Using a measuring tape, the circumference of the forearm was measured at its thickest point. We ensured that the forearm muscles were relaxed and uncontracted during measurements of circumference.

### 2.3. Experimental Protocol

**Figure 1:**
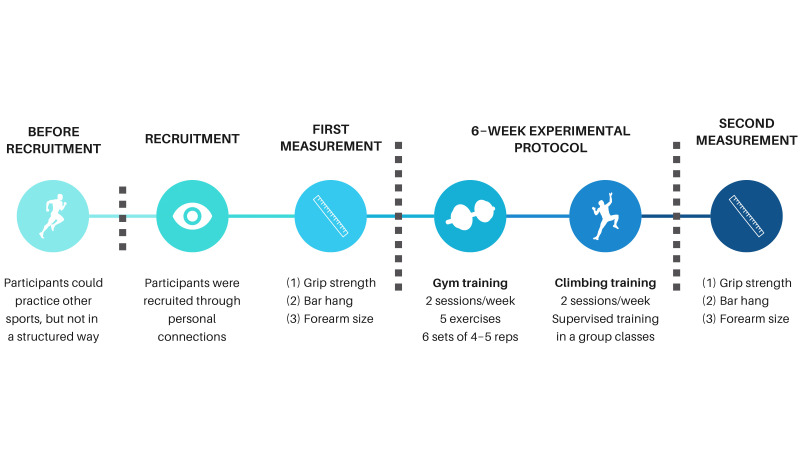
Experimental protocol.

During the 6-week protocol, participants had to follow climbing training two times per week, with gym workouts also two times per week. Participants were instructed to follow their standard diet as before the study during the experimental protocol. Subjects were instructed to report any missed workouts for any reason (delayed onset muscle soreness, injuries, lack of time, etc.). We did not receive any reports of non-compliance with the protocol, and all athletes completed the testing protocol in full agreement. We visualized the experimental protocol in Figure 1.

### 2.4. Gym and Climbing Training

The training intensity and weights were adjusted individually for each participant in consultation with the supervisor and assessed as “demanding but tolerable” at 75% of 1 rep max (corresponding to RPE ≈ 8) according to the guidelines from the American College of Sports Medicine [13]. A single gym workout should last approximately two hours. The exercises were performed in a stationary circuit format. Participants were instructed to perform each exercise separately and then move on to the next. The training began with a warm-up consisting of a few minutes of pre-programmed cardio work (walking on a treadmill, cycling without resistance, or elliptical) and basic exercises (arm circles, dynamic stretching). The training concluded with a few minutes of cool-down again on their preferred cardio machine and stretching. There were full-body workouts with focus on the muscles that have the greatest contribution to climbing performance (abdominal, arms, back, and chest exercises). Participants received instructions on how to properly perform all exercises and could contact the supervisor at any time during the study if they had any doubts regarding exercise technique. The workout consisted of upper-body exercises focused on grip strength and forearm muscles. The training consisted of 6 sets of 4–5 repetitions of each exercise: bench press, row, pull-ups, reverse bicep curl, and triceps pushdown. Participants could choose a machine, dumbbells, barbells, or cable pulleys, depending on their preferences. Our goal was to train specific muscle groups via comfortable exercises for the subjects. If the participant was unable to perform an exercise in a full form (e.g., pull-down), the participant could use a regression (e.g., lat pull-down machine). Participants could train at their preferred gym close to their place of residence, alone or with a friend.

Participants were instructed to attend group classes for climbing beginners at the Centre of Sport and Rehabilitation of the Medical University of Warsaw (Medical University of Warsaw, Warsaw, Poland). Training was supervised by a qualified climbing instructor and lasted about 1 h. The sessions consisted of (1) warm-up, (2) core training (climbing through dedicated pathways with assurance without pressure, climbing with a line, traversing), and (3) stretching and mobility work.

### 2.5. Study Population

There were 14 (56.0%) females and 11 (44.0%) males. Age was 20.4 ± 1.7 years, height was 173.8 ± 10.1 cm, and weight was 66.2 ± 10.7 kg. BMI of this population was 21.8 ± 1.7 kg·m^−2^. All the participants were beginners in climbing and had ≤1 week of experience. 4 (16.0%) participants did not practice any other type of sport whenever in their lives. 4 (16.0%) athletes practice 2 types of sport, and the remaining 17 (68.0%) trained in one other sport in the past. Table 1 presents demographic and anthropometric characteristics of the study population.

### 2.6. Statistical Analysis

We assessed the data distribution with the Shapiro–Wilk test and Q-Q plots. We reported the continuous data as mean and standard deviation due to a parametric distribution. Categorical data we presented as numbers and percentages. Comparison of results before and after the 6-week training period was performed using a paired Student’s *t*-test for dependent samples and presented with effect size (Cohen’s d for dependent means [d_z_]). We interpreted the effect size as low (d_z_ ≈ 0.2), moderate (d_z_ ≈ 0.5), and high (d_z_ ≈ 0.8) [19]. Additionally, we supported the differences and effect sizes with 95% confidence intervals (CI). We considered a two-sided *p*-value < 0.05 as significant. There were no missing data. We performed a post hoc population power assessment using G*Power (V3.1) for Student’s *t*-tests and a large effect size (d ≈ 0.8) [20]. The whole population achieved a power of 0.97, while males achieved a slightly too low power of 0.67, and females achieved a power at the verge of significance of 0.79.

All analyses were performed in SPSS (V30.0.0; IBM Corp., Armonk, NY, USA), and graphs were generated in GraphPad Prism (V10.0.0; GraphPad Software, Boston, MA, USA). Results were presented according to the 11th edition of the AMA *Manual of Style* guidelines.

## 3. Results

**Figure 2:**
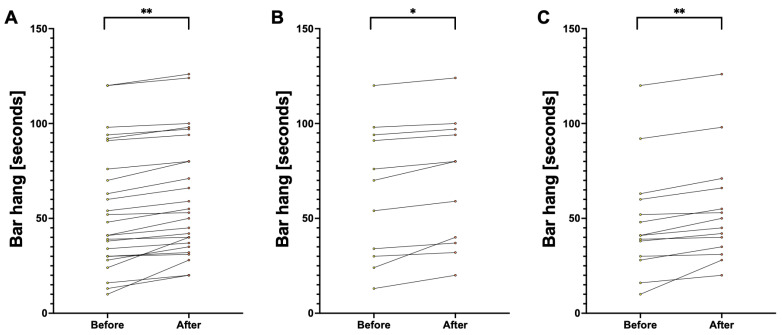
Comparison of hanging time before and after 6 weeks of training. Note: Each circle represents a separate participant, with a line representing within-subject change before and after the 6-week protocol. Panel (**A**) presents data for the whole population. Panel (**B**) presents data for males. Panel (**C**) presents data for females. Abbreviations: *, significant at *p* < 0.05; **, significant at *p* < 0.001.

**Figure 3:**
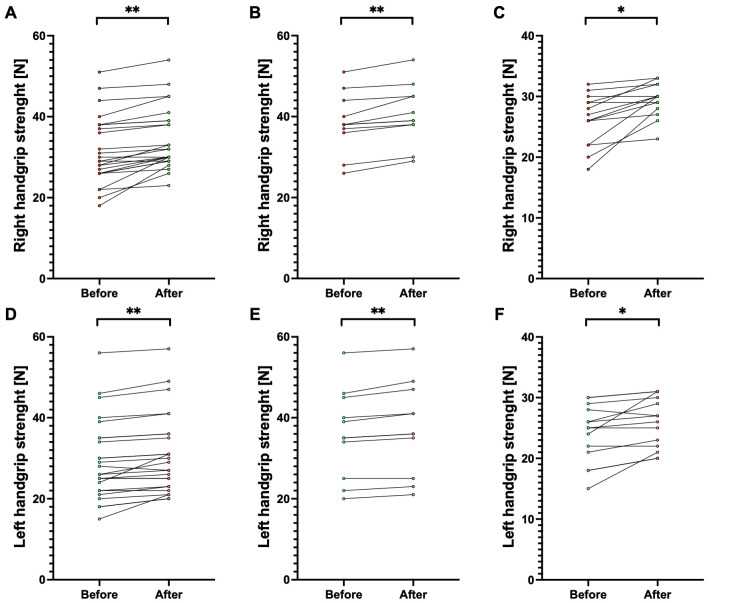
Comparison of handgrip strength before and after 6 weeks of training. Note: Each circle represents a separate participant, with a line representing within-subject change before and after the 6-week protocol. Panels (**A**,**D**) present data for the whole population. Panels (**B**,**E**) present data for males. Panels (**C**,**F**) present data for females. Abbreviations: *, significant at *p* < 0.05; **, significant at *p* < 0.001.

**Figure 4:**
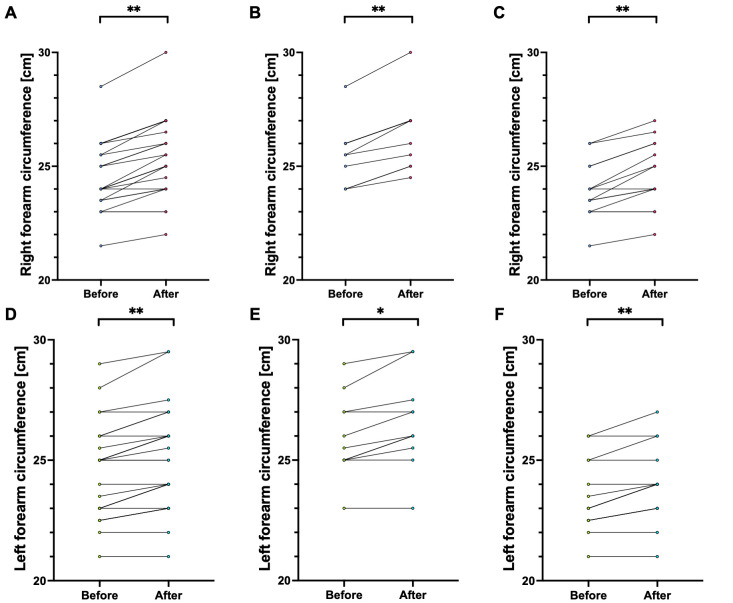
Comparison of forearm circumference before and after 6 weeks of training. Note: Each circle represents a separate participant, with a line representing within-subject change before and after the 6-week protocol. Panels (**A**,**D**) present data for the whole population. Panels (**B**,**E**) present data for males. Panels (**C**,**F**) present data for females. Abbreviations: *, significant at *p* < 0.05; **, significant at *p* < 0.001.

We presented the comparison of handgrip strength and muscle size in Table 2, both for the first and second assessments. We also presented data independently for each participant alongside within-subject change before and after the 6-week protocol on Figure 2 for handgrip time, Figure 3 for handgrip strength, and Figure 4 for forearm size.

Briefly, in each measurement, the participants noted an increase that was significant between the first and second assessments. Considering the whole population, all *p* < 0.001. Among males, all the measurements also increased between first and second assessment with *p* < 0.001, while for hanging time (64.0 ± 35.3 cm vs. 69.4 ± 33.7 cm; t(10) = 4.19, *p* = 0.002) and left forearm circumference (26.0 ± 1.7 cm vs. 26.5 ± 1.9 cm; t(10) = 3.99, *p* = 0.003) this relationship was only slightly less significant. Similarly, among females, each parameter increased with *p* < 0.001, and this relationship was slightly lower for right handgrip strength (26.1 ± 4.2 N vs. 29.4 ± 2.8 N; t(14) = 4.03, *p* = 0.001) and left handgrip strength (24.1 ± 4.7 N vs. 26.0 ± 4.2 N; t(14) = 3.14, *p* = 0.008).

The d_z_ ranged between 0.86 (0.23, 1.47) for left handgrip strength in females and 2.50 (1.26, 3.72) for right forearm circumference in males. In all measurements, d_z_ was > 0.8, indicating a large effect size.

## 4. Discussion

In this study, we found that 6 weeks of combined strength and climbing training is enough to induce significant progress in handgrip strength and muscle size. The results suggest that beginners could benefit from even shorter mesocycles than typically recommended, at least 8 weeks of training.

Six weeks of training is a period in which most amateurs expect to see noticeable results [11,21], despite the recommended training mesocycles being 8, 12, or 16 weeks [14]. Otherwise, already in the shorter training mesocycle, newcomers often become demotivated and abandon the activity [11]. The results of our study show that a 6-week period of intensive, structured training is sufficient to make significant progress. However, we implemented a combined protocol. Therefore, it would be interesting to see how those parameters change with separate training or climbing interventions.

Climbing is a popular sport among amateurs, as more and more climbing gyms and centers are being built where climbing is possible [5]. The social aspect of climbing with peers is also important [22]. Our study shows that amateur climbers should be especially motivated during the initial 6-week period. It should be emphasized that if amateurs notice progress in a short period of time, it is easier for them to become convinced of a given type of sport and remain consistent over the long term [23]. Strength training is currently one of the most popular sports among amateurs [24]. There are a significant number of public gyms available for training, and easy access to knowledge and supervisors (e.g., personal coaches) [25]. Therefore, we chose the combination of strength training and climbing, which is often practiced in real-world settings among amateurs and newcomers to the sport [5,26]. The practical implications of this study allow personal coaches and climbing instructors to develop motivational strategies to avoid discouraging athletes. Moreover, our study indicates that for newcomers, a 6-week training mesocycle could already make “true progress”. Fitness practitioners working with beginners may only program 6-week training mesocycles if their clients do not want to pay for a longer training program or only want to try whether they will like a particular training schedule or not.

Adaptations to strength training and the time at which muscle sizes increase among males and females may be different [14,25]. Typically, males will observe progress in strength and hypertrophy faster than females [14,25]. In our study, we saw that both the strength and muscle size were higher in males and females, which is consistent with other similar studies [27,28]. Moreover, all the relationships between measurements before and after the 6-week protocol are still significant. However, it is an important point for future research to recruit more participants, as the male subgroups achieved slightly too low power (0.67) and the female subgroup was on the verge of significance (0.79).

Diet and adequate caloric intake influence strength progress and muscle hypertrophy [29]. In our study, we recommended that the participants follow their standard diet. Most probably, all the participants eat a necessary amount of calories and nutrients. However, it is welcome for further studies to apply more objective dietary monitoring, perhaps a nutrition app or diary [30]. Additional evaluation of body composition would also be important.

At the same time, it is worth noting that in our study, we used a relatively intense protocol with several training sessions per week [13]. While this volume may be significant for amateurs, it could not be effective for more advanced athletes [31]. Typically, newcomers find it easier to make progress, and as they become more advanced, their gains slow [32]. This guide further researches participants with a higher performance caliber [31].

### Limitations

We did not consider different styles of climbing as OnSight and Flash, which have different levels of advancement [33]. The classification of participants and the description of their previous training experience based on self-reported data. The health status of the participants was also declared. To monitor compliance, we instructed the participants that they must report to the supervising researcher all omitted sessions for any reason (lack of time, injury, etc.). No participant reported the omissions, and all the subjects precisely fulfilled the protocol. However, we did not verify their compliance using objective methods, such as a training app or a training diary. We also did not require reporting the lifted weight, and the training was required to be subjectively demanding. We did not check body composition and only focused on BMI. Moreover, no one reported chronic fatigue, lack of energy, etc. The statistical power was on the verge of significance for females and too low for males. We also did not have a control group, which did not receive training or follow only a separate intervention (gym/climbing). We underscore the need to consider all the limitations and carefully interpret of presented results. We also recommend further studies on larger samples with a control group that assess the diet and training regimen more rigorously.

## 5. Conclusions

A 6-week training intervention consisting of structured climbing and strength training is sufficient to improve grip strength and the appearance of arm muscles in beginners. Amateurs should be motivated to train for at least 6 weeks to see clear progress, and could benefit from shorter mesocycles than typically recommended.

## Figures and Tables

**Table 1:** Characteristics of the population.

Variable		Whole Population (N = 25, 100.0%)	Males (N = 11, 44.0%)	Females (N = 14, 56.0%)
Age (years)		20.4 ± 1.7	20.2 ± 1.2	20.6 ± 2.0
Height (cm)		173.8 ± 10.1	183.5 ± 2.7	166.2 ± 6.5
Weight (kg)		66.2 ± 10.7	74.7 ± 7.5	59.5 ± 7.7
BMI (kg·m^−2^)		21.8 ± 1.7	22.2 ± 1.8	21.5 ± 1.7
Additional sport in the past	No other sports	4 (16.0%)		
	One other sport	17 (68.0%)		
	Two other sports	4 (16.0%)		
Training frequency in the past	Once per month	4 (16.0%)		
	Once per week	8 (32.0%)		
	Several Times per week	12 (48.0%)		
	Every day	1 (4.0%)		
Duration of single session	<1 h	5 (20.0%)		
	1 h	8 (32.0%)		
	>1 and <2 h	11 (44.0%)		
	≥2 h	1 (4.0%)		
Self-reported level of fitness	Very good	4 (16.0%)		
	Good	15 (60.0%)		
	Average	4 (16.0%)		
	Low	2 (8.0%)		
Warm-up before training	Yes	22 (88.0%)		
	No	3 (12.0%)		
Stretch after training	Yes	22 (88.0%)		
	No	3 (12.0%)		

Continuous data are presented as mean and standard deviation, and categorical data are presented as numbers and percentages.

**Table 2:** Comparison of handgrip strength and muscle size before and after 6 weeks of training.

Measured Variable	Before Training Intervention	After Training Intervention	Difference (95%CI)	*t*-Test (df)	Cohen’s d_z_ (95%CI)	*p*-Value
**Whole population (N = 25,100.0%)**						
Hanging time (seconds)	55.3 ± 3.2	60.9 ± 31.3	5.6 ± 4.2 (4.0, 7.3)	6.68 (24)	1.33 (0.77, 1.90)	<0.001
Right handgrip strength (N)	31.6 ± 8.4	34.3 ± 7.6	2.8 ± 2.5 (1.8, 3.7)	5.58 (24)	1.12 (0.59, 1.65)	<0.001
Left handgrip strength (N)	29.4 ± 9.9	31.0 ± 9.8	1.6 ± 1.7 (0.9, 2.3)	4.62 (24)	0.94 (0.46, 1.41)	<0.001
Right forearm circumference (cm)	24.6 ± 1.5	25.4 ± 1.7	0.8 ± 0.5 (0.6, 1.0)	9.04 (24)	1.60 (0.98, 2.22)	<0.001
Left forearm circumference (cm)	24.6 ± 2.0	25.1 ± 2.1	0.5 ± 0.4 (0.4, 0.7)	5.94 (24)	1.25 (0.72, 1.77)	<0.001
**Males (N = 11, 44.0%)**						
Hanging time (seconds)	64.0 ± 35.3	69.4 ± 33.7	5.4 ± 4.2 (2.9, 7.9)	4.19 (10)	1.29 (0.44, 2.05)	0.002
Right handgrip strength (N)	38.5 ± 7.3	40.5 ± 7.3	2.1 ± 1.3 (1.3, 2.9)	5.33 (10)	1.62 (0.68, 2.50)	<0.001
Left handgrip strength (N)	36.1 ± 10.9	37.4 ± 11.3	1.3 ± 0.8 (0.8, 1.7)	5.37 (10)	1.63 (0.69, 2.52)	<0.001
Right forearm circumference (cm)	25.5 ± 1.3	26.5 ± 1.5	1.0 ± 0.4 (0.7, 1.2)	9.04 (10)	2.50 (1.26, 3.72)	<0.001
Left forearm circumference (cm)	26.0 ± 1.7	26.5 ± 1.9	0.6 ± 0.5 (0.3, 0.9)	3.99 (10)	1.20 (0.40, 1.97)	0.003
**Females (N = 14, 56.0%)**						
Hanging time (seconds)	48.4 ± 29.1	54.3 ± 28.7	5.9 ± 4.3 (3.6, 8.1)	5.04 (14)	1.37 (0.62, 2.10)	<0.001
Right handgrip strength (N)	26.1 ± 4.2	29.4 ± 2.8	3.3 ± 3.0 (1.7, 4.9)	4.03 (14)	1.10 (0.42, 1.76)	0.001
Left handgrip strength (N)	24.1 ± 4.7	26.0 ± 4.2	1.9 ± 2.2 (0.7, 3.0)	3.14 (14)	0.86 (0.23, 1.47)	0.008
Right forearm circumference (cm)	24.0 ± 1.2	24.6 ± 1.5	0.7 ± 0.5 (0.4, 1.0)	5.26 (14)	1.40 (0.59, 2.21)	<0.001
Left forearm circumference (cm)	23.5 ± 1.5	24.0 ± 1.6	0.5 ± 0.4 (0.3, 0.7)	4.27 (14)	1.25 (0.53, 1.94)	<0.001

Note: Data are presented as mean and standard deviation. Abbreviations: df, degrees of freedom; 95%CI, 95% confidence interval; Cohen’s d_z_, Cohen’s d for dependent samples.

## Data Availability

The raw data supporting the conclusions of this article will be made available by the authors on request.

## Supplementary Materials

The following supporting information can be downloaded at: https://www.mdpi.com/article/10.3390/jfmk10040427/s1, Supplementary Material S1: STROBE checklist.
